# A Comparison Study of Growth Factor Expression following Treatment with Transcutaneous Electrical Nerve Stimulation, Saline Solution, Povidone-Iodine, and Lavender Oil in Wounds Healing

**DOI:** 10.1155/2013/361832

**Published:** 2013-06-03

**Authors:** Adalet Koca Kutlu, Dilek Çeçen, Seren Gülşen Gürgen, Oya Sayın, Ferihan Çetin

**Affiliations:** ^1^Manisa Health Sciences College, Celal Bayar University, 45020 Manisa, Turkey; ^2^Department of Histology and Embryology, School of Vocational Health Service, Celal Bayar University, Uncubozköy, 45010 Manisa, Turkey; ^3^Faculty of Medicine, Dokuz Eylül University Research Laboratory, 35340 Izmir, Turkey; ^4^Department of Physiology, Faculty of Medicine, İzmir University, Üçkuyular, 35340 Izmir, Turkey

## Abstract

This study compared the effects of transcutaneous electrical nerve stimulation (TENS), saline solution (SS), povidone-iodine (PI), and lavender oil (*Lavandula angustifolia*) through expression of growth factors in a rat model of wound healing. Six experimental groups were established, each containing 8 rats: a healthy group with no incision wounds, an incision-control group, an incision and TENS group, an incision and SS group, an incision and PI group, and an incision and lavender oil group. Experiments continued for 5 days, after which the skin in the excision area was removed. Tissue concentrations of epidermal growth factor (EGF) and platelet-derived growth factor (PDGF)-A were measured using enzyme-linked immunosorbent assay (ELISA). Tissue expressions of EGF, PDGF-A, and fibroblast growth factor (FGF)-2 were determined using immunohistochemistry. Wound closure progressed more rapidly in the TENS and lavender oil groups than in the control and other study groups. In particular, PDGF-A expressions in the dermis and EGF expression in the epidermis were significantly intense in the TENS group (*P* < 0.05). In addition, ELISA levels of growth factors such as PDGF-A and EGF were significantly higher in TENS group compared to the control group (*P* < 0.05). These immunohistochemical and ELISA results suggest that TENS may improve wound healing through increasing growth factors in the dermis and epidermis more than other topical applications.

## 1. Introduction

Wound healing is a process involving cellular, physiological, and biochemical events developing in response to tissue damage [1, 2]. The healing process has four phases: blood clotting, inflammation, new tissue formation, and tissue remodeling [3, 4]. A large number of growth factors and growth factor-binding proteins present in the epidermis and dermis are involved in wound healing [5]. These growth factors are expressed not only by damaged thrombocytes, endothelial basal membranes, and activated macrophages, but also by the epithelial cells [5, 6]. The most important of these growth factors are the epidermal growth factors (EGFs), insulin-like growth factors (IGFs), platelet-derived growth factors (PDGFs), and fibroblast growth factors (FGFs). These growth factors regulate cellular proliferation, differentiation and migration, and the synthesis of extracellular matrix proteins as well as angiogenesis during wound healing [5–7]. In addition, they are thought to regulate epithelial regeneration through stromal-epithelial paracrine interactions and direct receptors [5, 6, 8].

Increasing use of complementary medicines to treat a variety of conditions has led to growing interest in the potential of traditional and complementary methods for use in wound healing [9]. Transcutaneous electrical nerve stimulation (TENS), one of these methods, has been used to alleviate pain in humans and laboratory animals [10]. It was initially found to increase local blood flow in skin and mucous membrane damage by activating neural fibers and by regulating vascular resistance [11]. The electrical stimulation method has also been used for healing wounds in later studies [12, 13]. Another method employed for wound healing is the application of lavender oil. This is considered to be an alternative treatment option in wound healing because of its useful and effective biological activities [14, 15]. Due to its antimicrobial, anti-inflammatory, and analgesic properties, it is thought to prevent wound infections and to play a role in reducing pain by lowering inflammation [16].

Saline solution (SS) is a widely recommended irrigating and wound dressing agent, as it is compatible with human tissue [17, 18]. It causes no damage to new tissue and does not affect the functions of fibroblasts or keratinocytes in healing wounds [19].

PI promotes wound healing after surgical procedures and prevents contamination. Although it does not yield the best results, PI is used not only for the treatment of wounds, but also as a preoperative skin antisepsis treatment [20]. The long-term application of PI to large wounds is not recommended. In human studies, a cytotoxic effect has been observed against leukocytes, fibroblasts, and keratinocytes [21, 22].

The literature revealed no immunohistological and biochemical studies comparing the cellular differences between routinely used solutions and TENS or lavender oil during wound healing. The purpose of this study was therefore to evaluate the potential therapeutic effects of TENS and routine procedures on the PDGF-A, EGF, and FGF-2 expressions of cutaneous wound healing.

## 2. Materials and Methods

### 2.1. Animals

Twenty-four adult female and 24 adult male Wistar albino rats weighing approximately 150–200 g were used. All animal experiments were carried out in accordance with the European Communities Council Directive of November 24, 1986 (86/609/EEC), and were approved by the Animal Care Committee of Ege University. The animals were given a standard rodent chow diet and water ad libitum and kept at a constant temperature (22°C) in a light/dark cycle.

### 2.2. Experimental Procedure

Except for the healthy control group, all groups (2–6) were anesthetized with an intraperitoneal (i.p.) injection of ketamine (45 mg/kg) and xylazine (5 mg/kg). The dorsal surface was rinsed with 70% ethanol. After sterile preparation, an incision was made with a no. 21 scalpel blade on the dorsal surface of the rats. The wound (one per rat) consisted of an incision of a 1 cm full-thickness area of skin from the underlying fascia. The incision site was cleaned with SS and dried with sterile gauze after 15–20 s. The rats were kept in individual cages under a warming lamp and allowed to recover fully from anesthesia.

### 2.3. Treatment

The animals were divided into six groups: (1) a healthy group with no incision wounds (*n* = 8), (2) a control group with incision wounds (*n* = 8), (3) a group with incision wounds and treated with low intensity TENS (2 Hz, 15 min) (*n* = 8), (4) a group with incision wounds and treated with SS (*n* = 8), (5) a group with incision wounds and treated with PI (*n* = 8), and (6) a group with incision wounds and treated with lavender oil (*n* = 8).

In group 3, an AB-2100 electrical stimulation device (Able AB-2100, DC 4,5 V, 40 mA, Germany) was used for TENS; in group 4, SS (0.9% NaCl solution) was used; in group 5, PI (10% solution) was used, and in group 6, lavender oil (*Lavandula angustifolia* 0,882 g/mL density) was used with sterile sponges at 0.5 mL once a day for 5 days [23].

No treatment was applied to control animals' wounds, and all wounds remained uncovered during the experiment [16]. At the end of the 5th day, the rats were sacrificed and the skin at the excision sites was excised. A sample of skin was placed in 10% neutral formalin to be examined under a light microscope. In order to examine their biochemical parameters, homogenized skin samples were stored at −80°C.

### 2.4. Biochemical Examinations

#### 2.4.1. Homogenization of Rat Tissues for ELISA

All tissues were homogenized in 10 volumes of tissue extraction buffer. The homogenates were then centrifuged at 4000 rpm for 15 min. Supernatants' protein concentrations were determined using bicinchoninic acid (BCA) methods (Thermo, Rockford, IL, USA).

#### 2.4.2. PDGF-A and EGF Assays

Analysis of the PDGF-A (CUSABIO, limit determination 0.312 pg/mL-20 pg/mL) and EGF (OmniKine, limit determination 8 pg/mL-1000 pg/mL) levels in homogenized skin tissues was performed using ELISA kits. All assays were performed in accordance with the manufacturers' instructions. The plate was read on a Synergy HT, Multi-Detection Microplate Reader, BIO-TEK plate reader by measuring absorbance at a wavelength of 450 nm. All results are expressed as pg/mg protein.

### 2.5. Histological Examinations

The tissues were kept in 10% neutral formalin for 48 h. Once the tissue-blocking procedures were complete, 5 *μ*m serial sections from each skin block were placed on polylysine-coated slides.

After the serial sections had been deparaffinized and rehydrated, they were stored in a 0.5% trypsin solution (LabVision, USA) at room temperature for 15 min, following which they were treated with 3% H_2_O_2_ (LabVision, USA) for 5 min, in order to inhibit endogenous peroxidase activity in the tissue.

Some of the sections that had been washed 3 times with phosphate buffered saline were incubated with PDGF-A (Santacruz, USA) antibodies, and the rest with EGF (Santacruz, USA) and FGF-2 (Santacruz, USA) antibodies for 1 h. The sections were then washed 3 times with phosphate buffer solution, and biotinylated secondary antibody (Goat Anti-Rabbit, LabVision, USA) was applied. Thereafter, streptavidin peroxidase (Lab Vision, USA) was applied to the slides, and AEC (Lab Vision, USA) was used as a chromogen. Background staining was achieved with Mayer's hematoxylin. Preparate images were magnified under a CX41 bright-field microscope (Olympus, Tokyo, Japan) and recorded on a computer. Two independent observers, blinded to the treatment regimen, independently evaluated the immunolabeling scores. Labeling intensity was graded semiquantitatively, and the HSCORE was calculated using the formula HSCORE = Σ*Pi*(*i* + 1), where *i* is the intensity of labeling with a value of 1, 2, or 3 (weak, moderate, or strong, resp.) and *Pi* is the percentage of labeled epithelial and stromal cells for each intensity, within a range of 0–100% [24].

### 2.6. Statistical Analysis

Statistical analysis was performed on SPSS for Windows 11.5. Data were presented as the mean value ± standard deviation of the mean. Results were submitted to one-way ANOVA, followed by Tukey's post-test. Values of *P* < 0.05 were considered statistically significant.

## 3. Results

### 3.1. Macroscopic Investigation of Wound Healing

**Figure 1:**
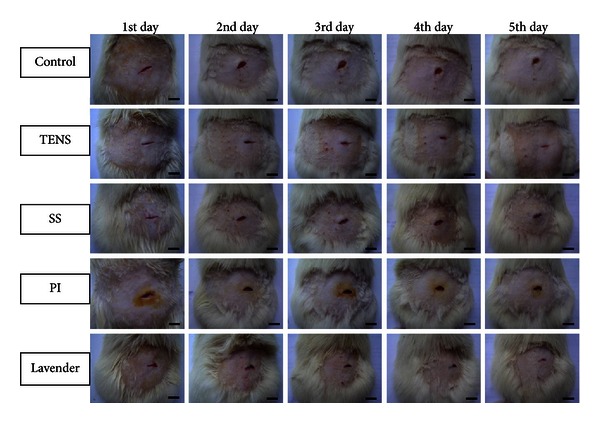
Macroscopic investigation of the wounds. Scale bars denote, 1 cm.

The effects of topical applied agents and TENS on wound healing were observed macroscopically during the 5-day wound-healing period in all 6 groups. No complication or infection occurred in any wounds (Figure 1).

In the control group, the wound edges were still distant from each other. Wound closure was noted to progress more rapidly in TENS-treated group compared to the other groups. In the SS group, wound healing was similar to that in the control group, but the wound edges were not close to each other. In the PI group, wound edges were further apart from each other than those in the other groups. In the lavender oil group, no signs of inflammation were observed in the wounds, and the wound edges were close to each other (Figure 1).

### 3.2. Biochemical Results

**Figure 2:**
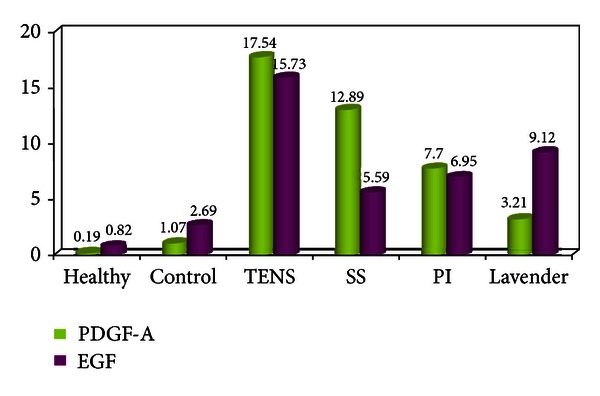
Means of PDGF-A and EGF ELISA values in the groups.

According to our ELISA results, the highest level of PDGF-A was in the TENS group (17.54 ± 1.08 pg/mL). The second highest levels of PDGF-A expression were in the SS (12.89 ± 0.82 pg/mL) and PI groups (7.70 ± 1.62 pg/mL) (*P* < 0.05). The healthy, control, and lavender oil groups exhibited lower PDGF-A expression levels (0.19 ± 0.09; 1.07 ± 0.54, and 3.21 ± 0.13 pg/mL resp.), and there was no statistically significant difference between these three. The TENS group had the highest EGF values (15.73 ± 0.76 pg/mL). The lowest EGF levels were in the healthy and control groups (0.82 ± 0.45 pg/mL and 2.69 ± 0.68 pg/mL, resp.). A moderate level of EGF was observed in the lavender oil, PI, and SS groups (9.12 ± 0.42; 6.95 ± 0.74; 5.59 ± 0.99 pg/mL) (Figure 2). PDGF-A values were significantly higher in all study groups compared to the control group at two-way comparison (*P* < 0.05). In terms of EGF levels, there was no statistically significant difference between the control and PI groups (*P* = 0.05) (Table 1).

### 3.3. Histological Examination of Wound Healing

**Figure 3:**
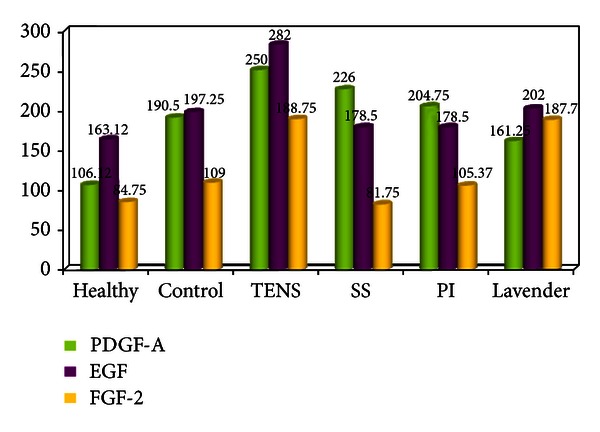
HSCORE values of PDGF-A, EGF, and FGF-2 of the groups.

**Figure 4:**
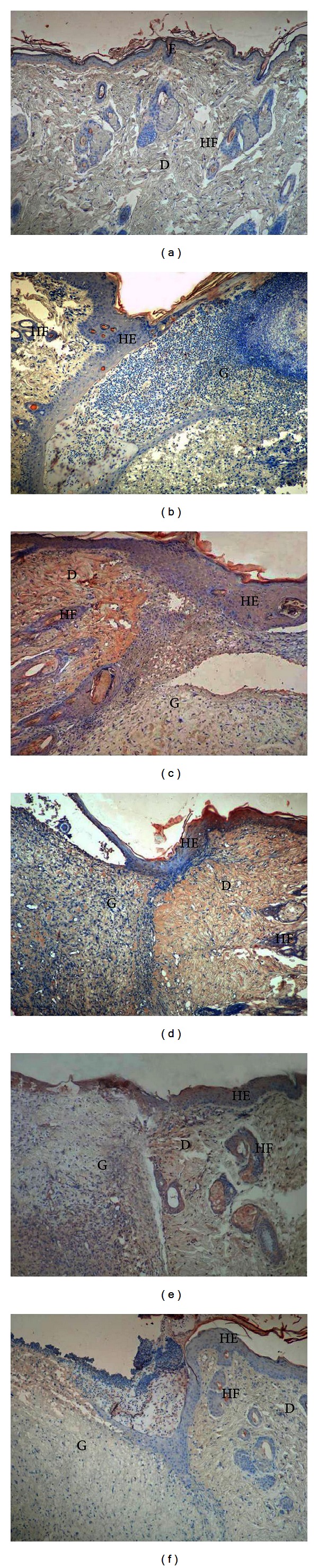
PDGF-A immunostaining of the skin tissues. Healthy group (a), control group (b), TENS group (c), SS group (d), PI group (e), and lavender oil group (f). E: epidermis, D: dermis, HF: hair follicles, HE: hyperproliferative epithelium, G: granulation tissue ×100.

PDGF-A immunoreaction in the healthy group was generally weak in all tissue (Figure 4(a)). A moderate reaction in the dermis and the epithelium was observed in the control group, and there was weak expression in granulation tissue in the wound area (190.50 ± 7.34). In addition, reepithelialization was not completed, and granulation tissue occupied a wide area (Figure 4(b)). In the TENS group, there was strong PDGF-A expression in the epithelium and dermis and moderate expression in granulation tissue (250.00 ± 9.79). Reepithelialization was rapid in the TENS group, and granulation tissue decreased significantly (Figure 4(c)). In the SS and PI groups, there was similar moderate immunoreaction in tissue generally (226.00 ± 9.79 and 204.75 ± 11.90, resp.). Epithelialization was weakly completed in both groups, and granulation tissue covered a wide area (Figures 4(d) and 4(e)). PDGF-A expression was weak in the lavender oil group, similar to that in the control group (161.25 ± 6.36). Additionally, epithelialization was partly completed and granulation decreased (Figure 4(f)). There was a significant difference (*P* < 0.05) between the groups in terms of mean PDGF-A values (Figure 3). At two-way comparison of the study groups' HSCORE PDGF-A levels with those of the control group, only the difference between the PI and control groups was not significant (*P* > 0.05) (Table 2).

**Figure 5:**
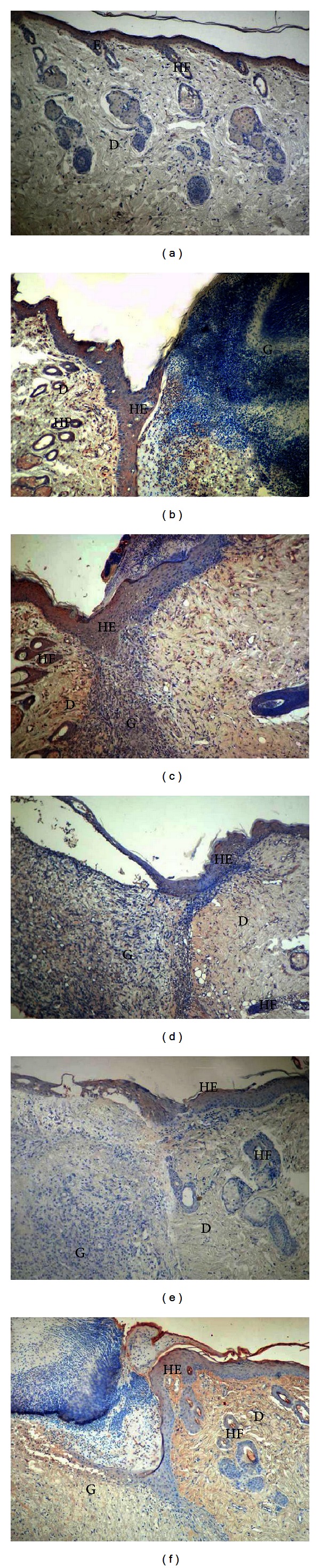
EGF immunostaining of the skin tissues. Healthy group (a), control group (b), TENS group (c), SS group (d), PI group (e), and lavender oil group (f). E: the epidermis, D: dermis, HF: hair follicles, HE: hyperproliferative epithelium, G: granulation tissue ×100.

In the healthy group, the EGF immunoreaction was weak in the dermis but weak to moderate in the epidermis and sebaceous glands (163.12 ± 6.97) (Figure 5(a)). Compared to the healthy group, increased reactivity was observed in the epidermis, dermis, and hair follicles in the control group (197.25 ± 6.96) (Figure 5(b)). A comparatively strong reaction was observed in the TENS group, particularly in the epithelium and hair follicles (282.00 ± 9.79) (Figure 5(c)). Decreased EGF staining was observed in the epidermis in the SS and PI groups (178.50 ± 7.34) and weak to moderate expression in the dermis and granular region (Figures 5(d) and 5(e)). The EGF immunoreaction in the lavender oil group was significantly greater (202.00 ± 9.79) than that in the SS and PI groups (178.50 ± 7.34), and hyperproliferation was moderate in the epidermis, hair follicles, and dermis (*P* < 0.05) (Figure 5(f)). However, no difference was determined at between the lavender oil and control groups at two-way comparison (*P* > 0.05) (Figure 3, Table 2).

**Figure 6:**
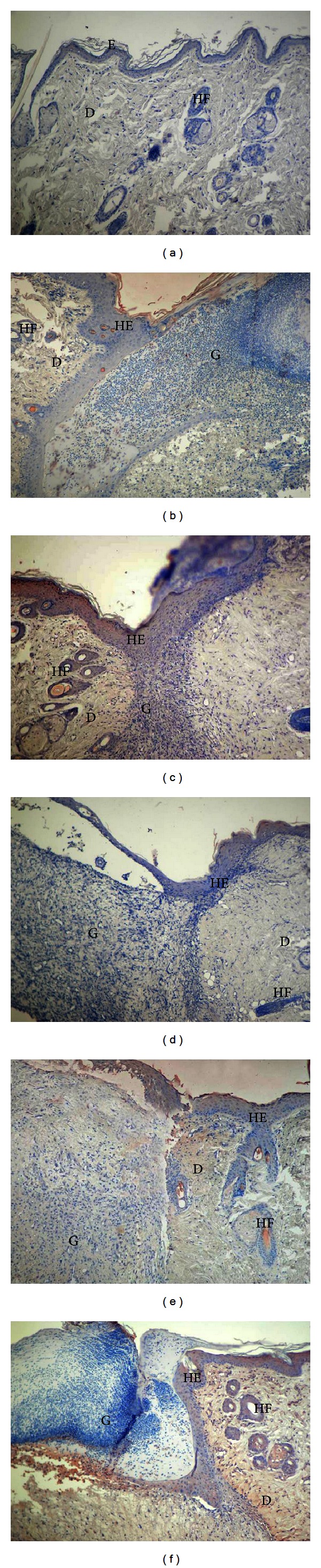
FGF-2 immunostaining of the skin tissues. Healthy group (a), control group (b), TENS group (c), SS group (d), PI group (e), the lavender oil group (f). E: epidermis, D: dermis, HF: hair follicles, HE: hyperproliferative epithelium, G: granulation tissue ×100.

Weak FGF-2 immunoreaction was determined in tissue in general in the healthy and SS groups (84.75 ± 5.11, 81.75 ± 4.06) (Figures 6(a)–6(d)). Weak to moderate expression was generally observed in the control and PI groups (109.00 ± 5.01, 105.37 ± 3.46) (Figures 6(b)–6(e)). However, the difference between the control and SS groups at two-way comparison was not significant (*P* > 0.05). FGF-2 expression was significantly higher in the epithelium and hair follicles and moderate in the dermis and granulation region in the TENS and lavender oil groups (188.75 ± 2.91 and 187.7 ± 3.90, resp.) compared to the control group (109.00 ± 5.01) (*P* < 0.05) (Figures 6(b)–6(f)) (Figure 3, Table 2).

## 4. Discussion

Wound healing is a coordinated process that includes cell proliferation, inflammation, collagen synthesis, and scar formation [2]. Various therapies are currently in use for treating open and chronic wounds. However, more efficient formulations are still required in cases of unsuccessful or deficient repair. We therefore evaluated and compared the role of TENS and other topical applications (PI, SS, and lavender oil) in the modulation of growth factors in the healing of cutaneous wounds.

*In vitro* studies have shown that electrical impulses increase the migration and proliferation of fibroblasts, the movement of macrophages, and phagocyte functions in wound healing [12, 25]. TENS also increases blood flow to wounds and fibroblasts induced by protein and DNA synthesis, reduces edema, and prevents bacterial growth. However, it has also been reported to have effects on epithelium and fibroblasts and to permit the migration of neutrophils and macrophages [10, 11, 26–28]. In this study, wound healing was significantly faster, and wound edges converged in rats treated with TENS compared to in the other groups.

Saline solution is a nonchemical material with no side-effects in the wound healing process [18]. Şelimen et al. reported that SS was the most appropriate solution, especially in the healing of surgical wounds [29]. In that study, wound healing in the SS group was similar to that in the control group, and the wound edges did not converge. Therefore, the macroscopic finding that SS does not accelerate wound healing but can be used safely in wound cleaning since it has no cytotoxic effects is consistent with the findings in the literature.

PI is widely used, especially in the dressing of surgical wounds. In human studies, PI has been shown to reduce infection rates and improve healing by reducing the bacterial load [20]. However, it is also reported that it is not necessary to use an antiseptic solution on clean wounds and that normal wound healing can be achieved by keeping the wound sufficiently moist with SS so long as aseptic principles are followed [21]. PI has been reported to have a cytotoxic effect, and it exhibits this by inhibiting tissue granulation, damaging endothelial cells, leukocytes, keratinocytes, and fibroblasts, and inactivating phagocytes, thus potentially delaying the healing process [21, 22]. Wound edges were further apart in the PI group than in the other groups in this study on rats. A delay in wound healing was observed macroscopically. The direct use of PI in surgical wounds is therefore not appropriate, and it should only be used to reduce the number of microorganisms in the wound surroundings.

The use of lavender oil in wound care is also recommended, but it is seldom used in health facilities. Lavender oil is known to have antibacterial, antifungal, sedative, and/or antidepressant effects and has traditionally been used. In addition to its antimicrobial effects, the anti-inflammatory analgesic properties of lavender oil have also been emphasized [14–16, 30, 31]. Macroscopic examination of the wound site in the lavender oil group rats in this study revealed no signs of edema, discharge, or local infection. These findings confirm the effects of lavender oil previously described in the literature. Lusby et al. reported that lavender oil and honey had positive effects on wound healing in rats [16]. The use of *L. Angustifolia* is particularly recommended in chronically infected wounds due to its immune-stimulating and antimicrobial effects. However, the efficacy of *L. Angustifolia* needs to be confirmed with randomized controlled trials in humans. There are also reports that lavender oil reduces scar tissue [30]. In their study of the cytotoxic effects of lavender oil components on fibroblasts and endothelial cells, Prashar et al. referred to the cytotoxic effect of linalyl acetate a component of lavender oil [32].

Injury of the skin triggers an extremely complex set of cellular and biochemical events, including inflammation, new tissue formation, and tissue remodeling, which finally lead to wound repair [2, 3]. The repair process, beginning immediately after injury, is mediated by various growth factors and cytokines released from the injured blood vessels and degranulating platelets [4–6].

The PDGF family is expressed by platelets, macrophages, vascular endothelium, fibroblasts, and keratinocytes and is a powerful mitogen for cells of mesenchymal origin, such as fibroblasts and smooth muscle cells. PDGF plays a role in every stage of wound healing [33]. In addition, PDGF plays an important role in the maturation of blood vessels [34]. It is also involved in reepithelialization by increasing the production of IGF-1 and thrombospondin-1 *in vitro*. Since PDGF increases the proliferation of fibroblasts, it also raises the production of extracellular matrix proteins. It also narrows collagen matrix and induces myofibroblasts in these cells because it stimulates fibroblasts. During tissue regeneration, PDGF contributes to the destruction of old collagens by matrix metalloproteinases [6]. PDGF-A is a PDGF subgroup. It is released from platelets and is the potential mitogen for all mesenchymal cells in wound healing. PDGF-A and TGF-*β*1 are the most potent stimulators of healing [35]. PDGF-A is a chemoattractant for inflammatory cells and fibroblasts and stimulates the synthesis of fibronectin, glycosaminoglycan, and collagenase [36]. PDGF-A also induces the production of matrix metalloproteinases through fibroblasts and myofibroblasts. This enzyme leads to the contraction of matrix collagens and production of vascular endothelial growth factor which contributes to the formation of new capillaries [37]. PDGF-A expression in our healthy group was weak, while the fact that this increased to some extent in the epithelium and dermis in the control group demonstrated that this growth factor stimulated fibroblasts in wound healing and therefore played a role in dermis repair. PDGF-A exhibiting stronger reaction in the dermis and epithelial tissue in the TENS group compared to other topical treatments according to our immunohistochemical and biochemical findings suggested that electrical stimulation is more effective in activating fibroblasts in rebuilding the dermis.

The EGF family is one of the most well-defined growth factor families, playing a significant role in wound healing. EGF binds to the EGF receptor, a tyrosine kinase transmembrane protein, resulting in the dimerization and autophosphorylation of the receptor and the tyrosine phosphorylation of the proteins [6]. EGF is released by platelets, macrophages, and fibroblasts and has a paracrine effect on keratinocyte [2]. *In vitro* studies indicate that EGF is released in high quantities after acute injury and increases reepithelialization and wound resistance to stretching [2]. According to several studies, the expression of EGF in a wound site is highest during active epithelialization and wound contraction. EGF stimulates granulation tissue formation both *in vitro* and *in vivo* [2].

FGFs are important signaling molecules that play a key role in growth and morphogenesis as well as in many physiological and pathological conditions, such as wound healing, neovascularization, and tumor growth. FGFs induce mitogenic, angiogenic, and chemotaxic activities in a variety of different cell types. In addition to their mitogenic activities, FGFs are key activators of tumor-induced angiogenesis. FGF-2, or basic FGF, a member of the FGF family, plays a role in the granulation of tissue formation, angiogenesis, reepithelialization, and tissue renewal (regeneration) during acute wound healing. FGF-2 regulates the synthesis and accumulation of various extracellular matrix proteins and increases the migration of keratinocytes during reepithelialization [38]. FGF-2 is produced by macrophages and endothelial cells [2] and is upregulated by thrombin and by itself [39]. In this study, reepithelialization was at the highest level in our histological findings in the TENS and lavender oil groups, parallel to the macroscopic findings. According to our immunohistochemical and ELISA findings, EGF and FGF-2 reactions were again highest in the TENS and lavender oil groups, from which we concluded that these two procedures were effective in the stimulation of reepithelialization and granulation tissue formation.

## 5. Conclusion

We think that TENS increases dermis renewal and contraction through PDGF-A, the most potent stimulator, in wound healing. We also concluded that it strengthens reepithelialization and permits a decrease in granulation tissue by also stimulating EGF and FGF-2 expressions. Lavender oil would seem to accelerate reepithelialization and wound closure by enhancing EGF secretion. However, since it has no effect on PDGF-A release, it is not very effective in dermis renewal and therefore does not accelerate wound healing as much as TENS. SS and PI, widely used techniques in wound closure, had a more positive effect than the on wound healing compared to the control group by increasing moderate expressions of the growth factors PDGF-A, EGF, and FGF-2.

In conclusion, TENS accelerates wound healing more than SS, PI, and lavender oil, other wound treatment methods used, by increasing growth factors in the tissue. These results suggest that if TENS is to be used in postoperative surgical wounds, this should be supported with experimental studies conducted with humans.

## Figures and Tables

**Table 1:** Examination of mean PDGF-A and EGF ELISA values in the groups and two-way comparisons of control and other groups.

	Control	TENS	PI	SS	Lavender oil	*P* value			
						C-TENS	C-PI	C-SS	C-LO
PDGF-A	1.07 ± 0.54	17.54 ± 1.08	7.70 ± 1.62	12.89 ± 0.82	3.21 ± 0.13	0.000	0.000	0.000	0.000
EGF	2.69 ± 0.68	15.73 ± 0.76	6.95 ± 0.74	5.59 ± 0.99	9.12 ± 0.42	0.000	0.05*	0.000	0.000

TENS: transcutaneous nerve stimulation; PI: povidone-iodine; SS: saline solution; C: control; LO: lavender oil; *P < 0.05 one-way analysis of variance ANOVA, data are expressed as mean ± SD.

**Table 2:** Examination of mean PDGF-A, EGF, and FGF-2 immunohistochemistry HSCORE values in the groups and two-way comparisons of control and other groups.

	Control	TENS	PI	SS	Lavender oil	*P* value			
						C-TENS	C-PI	C-SS	C-LO
PDGF-A	190.50 ± 7.34	250.00 ± 9.79	204.75 ± 11.90	226.00 ± 9.79	161.25 ± 6.36	0.000	0.330*	0.000	0.000
EGF	197.25 ± 6.96	282.00 ± 9.79	178.50 ± 7.34	178.50 ± 7.34	202.00 ± 9.79	0.000	0.001	0.001	0.849*
FGF-2	109.00 ± 5.01	188.75 ± 2.91	105.37 ± 3.46	81.75 ± 4.06	187.7 ± 3.90	0.000	0.001	0.409*	0.000

TENS: transcutaneous nerve stimulation; PI: povidone-iodine; SS: saline solution; C: control; LO: lavender oil; *P < 0.05 one-way analysis of variance ANOVA, data are expressed as mean ± SD.
